# Evaluation of the effects of motion mitigation strategies on respiration‐induced motion in each pancreatic region using cine‐magnetic resonance imaging

**DOI:** 10.1002/acm2.12693

**Published:** 2019-08-05

**Authors:** Koya Fujimoto, Takehiro Shiinoki, Yuki Yuasa, Ryota Onizuka, Masatoshi Yamane

**Affiliations:** ^1^ Department of Radiation Oncology, Graduate School of Medicine Yamaguchi University Yamaguchi Japan; ^2^ Department of Radiological Technology Yamaguchi University Hospital Yamaguchi Japan

**Keywords:** cine‐magnetic resonance imaging, motion mitigation strategies, pancreas, respiration‐induced motion

## Abstract

**Purpose:**

This study aimed to quantify the respiration‐induced motion in each pancreatic region during motion mitigation strategies and to characterize the correlations between this motion and that of the surrogate signals in cine‐magnetic resonance imaging (MRI). We also aimed to evaluate the effects of these motion mitigation strategies in each pancreatic region.

**Methods:**

Sagittal and coronal two‐dimensional cine‐MR images were obtained in 11 healthy volunteers, eight of whom also underwent imaging with abdominal compression (AC). For each pancreatic region, the magnitude of pancreatic motion with and without motion mitigation and the positional error between the actual and predicted pancreas motion based on surrogate signals were evaluated.

**Results:**

The magnitude of pancreatic motion with and without AC in the left–right (LR) and superior–inferior (SI) directions varied depending on the pancreatic region. In respiratory gating (RG) assessments based on a surrogate signal, although the correlation was reasonable, the positional error was large in the pancreatic tail region. Furthermore, motion mitigation in the anterior‐posterior and SI directions with RG was more effective than was that with AC in the head region.

**Conclusions:**

This study revealed pancreatic region‐dependent variations in respiration‐induced motion and their effects on motion mitigation outcomes during AC or RG. The magnitude of pancreatic motion with or without AC and the magnitude of the positional error with RG varied depending on the pancreatic region. Therefore, during radiation therapy for pancreatic cancer, it is important to consider that the effects of motion mitigation during AC or RG may differ depending on the pancreatic region.

## 1 INTRODUCTION

Pancreatic cancer is a malignant disease with high mortality. Although surgery is the standard treatment for this condition, most patients have locally advanced unresectable disease at the time of diagnosis and are therefore unable to undergo curative resection.^1^ Hence, chemoradiation is an integral part of treatment for these patients.^2^, ^3^

Dose escalation in pancreatic cancer has attracted attention due to the high radiation resistance of locally advanced pancreatic cancer^4^, ^5^. In a recent report,^6^ it was suggested that high‐dose adaptive radiation therapy improves the overall survival of patients with pancreatic cancer. However, it is difficult to deliver sufficiently large doses because the pancreas is adjacent to multiple organs at risk (OARs), including highly radiosensitive organs such as the stomach and duodenum.^7^, ^8^ Intensity‐modulated radiation therapy for pancreatic cancer is an effective strategy that allows hypofractionated high‐dose radiotherapy while sparing the OARs.^9^, ^10^ However, radiation delivery for pancreatic cancer is also complicated by respiration‐induced motion of the pancreas, which has been evaluated previously using four‐dimensional computed tomography (4DCT),^11^, ^12^ cone‐beam computed tomography (CBCT),^13^ and cine‐magnetic resonance imaging (cine‐MRI).^14^, ^15^

The efficacy of motion mitigation strategies for lung or abdominal tumor radiotherapy has been evaluated using abdominal compression (AC), tumor tracking, or respiratory gating (RG) strategies based on the surrogate signal (abdominal wall or implanted fiducial marker).^13^, ^16^, ^17^, ^18^, ^19^, ^20^ For instance, Campbell et al.^13^ evaluated the efficacy of motion mitigation strategies for pancreatic radiotherapy using CBCT. The authors reported that RG based on an abdominal wall surrogate enables greater tumor motion mitigation than does that with AC. However, Feng et al.^14^ reported that the pancreatic tumor border position does not correlate well with the abdominal wall or diaphragm position. In addition, it has been demonstrated that 4DCT or CBCT underestimates the internal target volume of the tumor.^21^, ^22^ Furthermore, Fernandes et al.^22^ reported large differences between liver tumor motion measured using 4DCT and that measured using cine‐MRI. Thus, the evaluation of respiration‐induced pancreatic motion using CBCT by Campbell et al.^13^ may not have been adequate, and further detailed evaluation using MRI is required.

It is known that MRI‐guided radiotherapy system enables excellent soft tissue visualization and real‐time direct tracking of respiratory tumor motion using cine‐MRI during treatment.^23^, ^24^ Thus, there is increased interest in detailed quantification of respiration‐induced tumor motion and evaluation of the efficacy of motion mitigation strategies using MR images.^15^, ^18^, ^25^ To our knowledge, few studies have reported the magnitude of respiration‐induced motion in each pancreatic region with AC by employing cine‐MRI. Furthermore, no study has reported the relationship between the respiration‐induced motion in each pancreatic region and that in the abdominal wall surrogates.

Therefore, this study aimed to quantify the magnitude of respiration‐induced motion in each pancreatic region with and without AC and to quantify the positional error between actual and predicted pancreas motion on the basis of RG strategies using cine‐MRI. We also aimed to quantify the effects of pancreatic region‐dependent variations in respiration‐induced motion on the outcomes of motion mitigation strategies during AC or RG.

## 2 MATERIALS AND METHODS

### 2.1 Image acquisition

**Figure 1:**
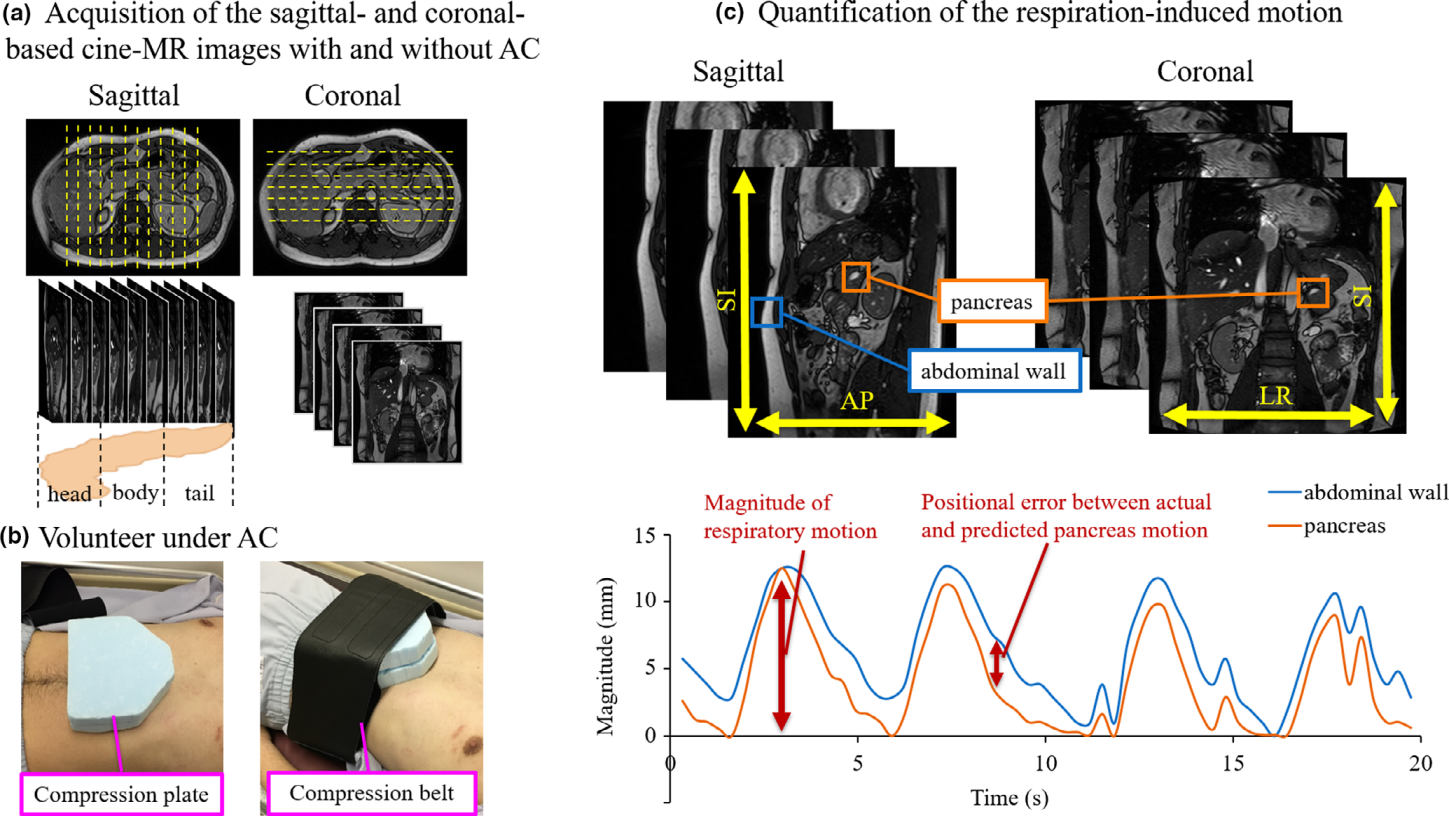
Workflow of the evaluation method. (a) Acquisition of the sagittal‐ and coronal‐based cine‐MR images with and without abdominal compression (AC). (b) Volunteer positioning under AC. (c) Respiration‐induced motion acquisition of the pancreas and surrogate signal (abdominal wall motion). In each slice, template images of the pancreas (orange) and abdominal wall (blue) were obtained at the end‐exhalation phase. Following this, respiration‐induced motion signals were obtained by the template‐matching technique.

Eleven healthy volunteers (mean age: 33 yr, range: 25–59 yr) were enrolled in this study. Each volunteer under fasting for at least five hours underwent basic respiratory training before image acquisition. Sagittal‐ and coronal‐based cine‐MR images were obtained using the TrueFISP sequence (balanced steady‐state free precession sequence) without motion mitigation in 11 volunteers, and eight of these volunteers also underwent image acquisition under AC with the following acquisition parameters: repetition time = 3.3 ms, echo time = 1.6 ms, flip angle = 49.0°, slice thickness = 3.0 mm, spatial resolution = 0.9 × 0.9 mm, and bandwidth = 1370.0 Hz/pixel [Fig. 1(a)]. AC was performed using an in‐house‐developed compression device that was applied to the subxiphoid area during each volunteer's end‐expiration until it reached maximum tolerability [Fig. 1(b)]. Cine‐MR images were obtained at 3.3 Hz for 20 s using a 3.0‐Tesla MRI scanner (MAGNETOM Prisma, Siemens, Germany) with a 16‐channel phased array coil. Scanning was performed with the volunteers under audio instructions (5–6 s period) in the supine position. Institutional review board approval was obtained, and all volunteers provided informed consent prior to study participation.

### 2.2 Quantification of respiration‐induced pancreatic motion with and without AC

The workflow for quantifying respiration‐induced motion is shown in Fig. 1(c). To obtain template images for quantifying respiration‐induced pancreatic motion, in each slice of cine‐MRI, regions of interest (ROIs) were set at the pancreas in images of the end‐exhalation phase with and without AC. The ROIs were set in the vessels adjacent to the pancreas (head region: portal vein, body and tail region: splenic vein). Following this, respiration‐induced motion signals for each organ were obtained by a template‐matching algorithm implemented in MATLAB (Version: R2016a, MathWorks, Natick, MA).^26^, ^27^ The respiration‐induced motion signals of the pancreas in the anterior–posterior (AP) and superior–inferior (SI) directions were obtained in the sagittal cine‐MR images and those in the left–right (LR) and SI directions were obtained in the coronal cine‐MR images. To evaluate the magnitude of the respiration‐induced motion in each region, maximal motions were calculated for all acquired slices of the sagittal and coronal cine‐MRI datasets and averaged for each pancreatic region.

### 2.3 Quantification of the correlation and the positional error between the actual and predicted pancreas position based on the surrogate signal

In each slice of the sagittal cine‐MR images without motion mitigation, the ROI was set at the pancreas, and the abdominal wall was defined as the region 5.0 cm below the xiphoid process at the end‐exhalation phase. Respiration‐induced motion of the abdominal wall in the AP direction was then obtained [Fig. 1(c)]. The predicted motions of the pancreas based on the surrogate signal in AP and SI directions were calculated by multiplying the relative abdominal wall motion in AP direction and the magnitude of the actual pancreatic motion in AP and SI directions respectively. The Pearson’s correlation coefficients between the actual and predicted pancreas motion based on the abdominal wall surrogate were calculated for the sagittal cine‐MRI datasets in the linear relationship of displacement along the acquisition time (20 s) using Matlab software. The averaged positional errors along the acquisition time between the actual and the predicted pancreas motion based on the abdominal wall surrogate were calculated for all acquired slices in the sagittal datasets and averaged for each pancreatic region.

### 2.4 Data analysis

Evaluations of the following items were performed for each pancreatic region: (a) magnitude of the respiration‐induced pancreatic motion with and without AC in the LR, AP, and SI directions; (b) correlation and predicted positional error between actual pancreas motion (AP and SI directions) and predicted pancreas motion based on the abdominal wall surrogate in the AP direction; and (c) motion mitigation by using AC and RG with the abdominal wall surrogate on the basis of the abdominal wall motion. In the LR, AP, and SI directions, the differences in the magnitude of respiration‐induced pancreatic motion with and without AC were calculated. To simulate RG when using an abdominal wall surrogate, the duty cycle was defined as 40% of the typical beam duty cycle values.^28^, ^29^ Therefore, for each volunteer, the maximum displacement of the pancreas between end‐expiration (50% phase) and either the 30% or the 70% phase was calculated, as described in previous studies.^13^, ^30^

These results were averaged for each pancreatic region, and the differences with the magnitude of the pancreatic motion without mitigation were calculated. In the analysis of RG, sagittal‐based cine‐MRI datasets were used to evaluate respiration‐induced motion in the AP and SI directions at the same time phase.

Tukey’s Honestly Significant Difference test^31^ was used to analyze the differences in the magnitude of respiration‐induced motion and the predicted positional error between the pancreatic regions. The paired *t*‐test was used to compare differences in the magnitude of motion mitigation between the AC and RG conditions. Data analyses were performed using Matlab software. The level of statistical significance was set at *P* < 0.05.

## 3 RESULTS

### 3.1 Magnitude of respiration‐induced pancreatic motion with and without AC

**Figure 2:**
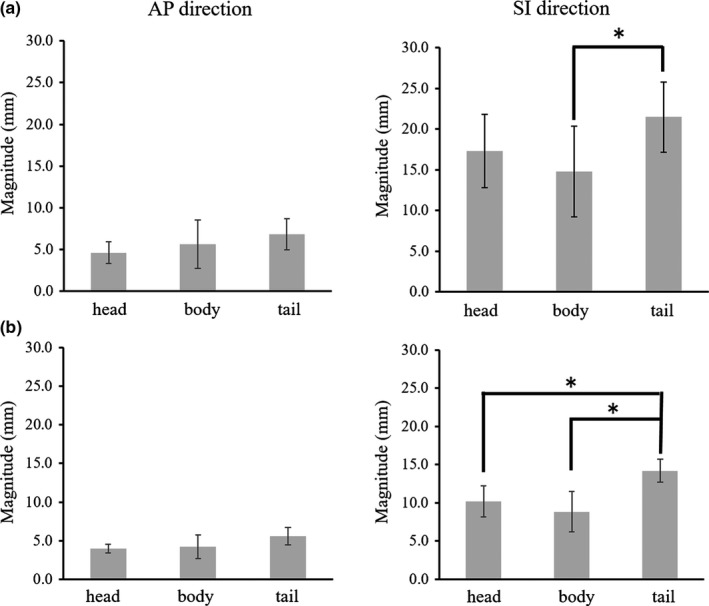
The magnitude of pancreatic motion in the anterior–posterior (AP), and superior–inferior (SI) directions obtained in the sagittal cine‐MR images. (a) Without motion mitigation. (b) With abdominal compression. *P < 0.05.

**Figure 3:**
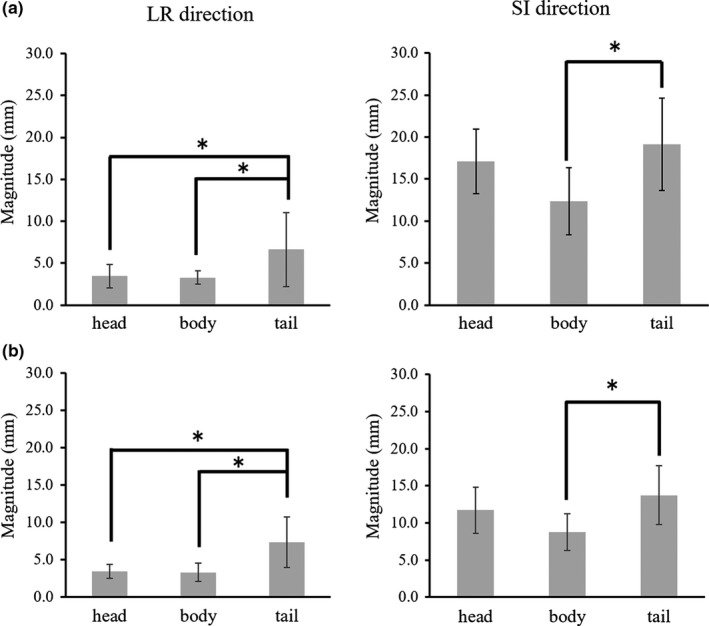
The magnitude of pancreatic motion in the left–right (LR) and superior–inferior (SI) directions obtained in the coronal cine‐MR images. (a) Without motion mitigation. (b) With abdominal compression. *P < 0.05.

Table 1 shows the magnitude of respiration‐induced motion in each pancreatic region in the LR, AP, and SI directions without motion mitigation in 11 volunteers and in eight of these volunteers under AC. Figure 2 shows the magnitude of respiration‐induced motion in each pancreatic region in the AP and SI directions obtained in the sagittal cine‐MR images without motion mitigation (a) and with AC (b). In the SI direction without AC, the magnitude of pancreatic motion was significantly greater in the tail region than in the body regions (*P* = 0.011). In the SI direction with AC, the magnitude of pancreatic motion was significantly greater in the tail region than in the other regions (head to tail: *P* = 0.025, body to tail: *P* = 0.001). Figure 3 shows the magnitude of respiration‐induced motion in each pancreatic region in the LR and SI directions obtained in the coronal cine‐MR images without motion mitigation (a) and with AC (b). In the LR direction, both with and without AC, the magnitude of pancreatic motion was significantly greater in the tail region than in the other regions (head to tail: *P* = 0.008, body to tail: *P* = 0.006 and head to tail: *P* = 0.037, body to tail: *P* = 0.025, respectively). In the SI direction without AC, the magnitude of pancreatic motion was significantly greater in the tail region than in the body regions (*P* = 0.004). In the SI direction with AC, the magnitude of pancreatic motion was significantly greater in the tail region than in the body region (*P* = 0.01).

**Table 1:** Magnitude of pancreatic motion in the left–right (LR), anterior–posterior (AP), and superior–inferior (SI) directions in 11 volunteers without motion mitigation and in eight with abdominal compression (AC) which were obtained in coronal and sagittal cine‐MR images.

Volunteer No.	Head (without AC/with AC)				Body (without AC/with AC)				Tail (without AC/with AC)			
	Coronal		Sagittal		Coronal		Sagittal		Coronal		Sagittal	
	LR (mm)	SI (mm)	AP (mm)	SI (mm)	LR (mm)	SI (mm)	AP (mm)	SI (mm)	LR (mm)	SI (mm)	AP (mm)	SI (mm)
1	3.6/2.7	18.2/13.7	4.9/4.9	13.1/10.0	2.7/3.6	15.5/12.8	4.0/2.4	11.5/9.0	4.6/6.8	20.1/18.2	6.8/4.9	18.8/14.9
2	2.3/4.1	15.5/11.8	3.3/4.9	14.3/8.8	3.6/3.4	12.4/7.7	3.0/5.8	12.2/7.3	4.1/3.6	14.1/10.3	4.6/3.8	21.6/13.4
3	5.5/–	26.4/–	4.9/–	17.5/–	3.2/–	7.3/–	3.0/–	11.6/–	4.6/–	29.2/–	8.3/–	24.5/–
4	3.8/3.1	14.8/8.5	4.0/3.8	14.5/8.3	3.1/2.6	11.0/7.4	6.3/4.5	10.7/7.0	8.3/8.0	20.3/13.3	7.0/4.8	20.5/13.0
5	6.8/4.1	21.0/12.8	5.4/3.1	20.4/13.0	4.6/2.3	20.8/12.7	13.4/6.9	25.2/13.7	11.8/14.9	26.0/20.7	11.2/7.0	30.5/16.6
6	2.7/1.8	18.2/4.6	4.3/3.9	10.6/8.2	2.3/0.9	15.5/5.5	3.5/2.6	14.4/6.1	1.4/4.6	16.4/10.0	4.9/5.5	14.2/13.0
7	3.0/–	17.3/–	7.2/–	26.0/–	2.7/–	12.3/–	7.6/–	25.2/–	4.6/–	20.5/–	6.2/–	27.6/–
8	1.8/–	14.6/–	1.8/–	14.3/–	2.7/–	5.5/–	3.8/–	11.8/–	1.8/–	9.1/–	4.5/–	20.2/–
9	3.2/2.7	16.0/10.9	5.8/3.8	23.1/14.0	5.0/3.6	14.1/6.4	6.5/4.7	17.1/7.9	14.6/9.6	22.3/12.8	7.8/7.6	20.2/15.1
10	2.7/3.6	15.0/14.1	4.6/1.4	20.7/14.0	3.2/5.0	10.5/10.4	6.2/1.4	17.2/12.8	10.5/4.6	13.2/8.7	5.8/1.0	18.1/15.6
11	2.7/5.0	15.9/10.9	4.6/3.6	15.6/10.0	2.7/4.6	10.9/10.6	4.2/2.4	12.8/7.0	0.9/6.4	19.1/15.5	7.6/5.2	19.9/11.8
Mean	3.5/3.4	17.1/10.9	4.6/3.7	17.3/10.2	3.3/3.3	12.3/9.2	5.6/3.9	14.8/8.8	6.1/7.3	19.1/13.7	6.8/5.0	21.5/14.2

On comparison of the magnitude of respiration‐induced motion in the entire pancreatic region with and without AC, the application of AC significantly mitigated respiration‐induced pancreatic motion in the AP and SI directions (AP, *P* = 0.03; SI, *P* = 0.0004). However, AC did not significantly mitigate the pancreatic motion in the LR direction (*P* = 0.36). Furthermore, pancreatic region‐dependent variations in magnitude of the respiration‐induced motion were observed regardless of the use of AC.

### 3.2 Correlation and positional error between actual and predicted pancreas motion

The Pearson’s correlation coefficients between abdominal wall motion and pancreatic motion, which were averaged in the head, body, and tail regions, were 0.88 (range, 0.80–0.96), 0.83 (range, 0.48–0.94), and 0.94 (range, 0.78–0.97), respectively, in the AP direction and 0.95 (range, 0.84–0.99), 0.95 (range, 0.90–0.98), and 0.97 (range, 0.91–0.99), respectively, in the SI direction.

**Figure 4:**
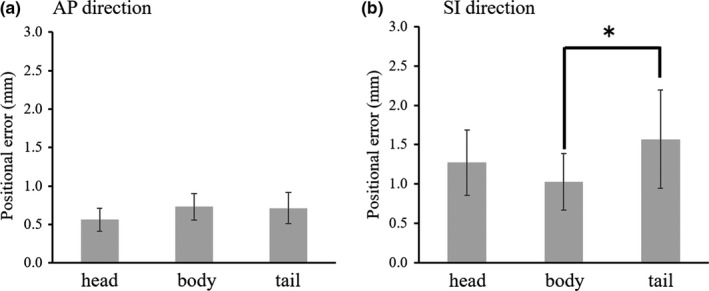
Positional errors between the actual and predicted pancreas motion based on the abdominal wall surrogate in the anterior–posterior (AP) (a) and superior–inferior (SI) directions (b). *P < 0.05.

Figure 4 shows the positional errors between the actual and predicted pancreas motion based on the abdominal wall surrogate in the AP (a) and SI directions (b). The magnitude of the predicted positional error in the SI direction differed between the body and tail regions (*P* = 0.04). The maximal error among all the volunteers was found to be 2.9 mm in the tail region.

### 3.3 Motion mitigation with AC and RG

Table 2 shows the maximum displacement of the pancreas between end‐expiration (50% phase) and either the 30% or the 70% phase in each pancreatic region when simulating RG based on an abdominal wall surrogate (duty cycle: 40%). In the SI direction, the maximum displacement of the pancreas between end‐expiration (50% phase) and either the 30% or 70% phase was significantly greater in the tail region than in the other regions (head to tail: *P* = 0.0002, body to tail: *P* = 0.0003).

**Table 2:** Maximum displacement of the pancreas between end‐expiration (50% phase) and either the 30% or the 70% phase when simulating the respiratory gate based on an abdominal wall surrogate (duty cycle: 40%) in the anterior–posterior (AP) and superior–inferior (SI) directions obtained in sagittal cine‐MRI images in 11 volunteers.

Volunteer no.	Head		Body		Tail	
	AP (mm)	SI (mm)	AP (mm)	SI (mm)	AP (mm)	SI (mm)
1	3.6	8.5	2.3	6.4	3.3	10.3
2	2.7	8.7	2.4	7.9	3.0	16.1
3	1.8	8.7	2.1	7.6	5.5	16.0
4	2.5	7.8	2.2	7.1	2.7	14.5
5	4.2	8.9	4.3	9.4	9.6	17.8
6	1.4	4.6	1.8	3.9	2.3	7.0
7	3.0	7.7	6.4	9.6	3.9	15.8
8	1.2	8.2	2.1	6.4	2.3	13.2
9	2.7	9.4	3.2	14.1	6.4	14.6
10	2.4	9.7	5.9	10.5	4.9	9.1
11	1.8	6.6	2.3	6.6	4.1	10.9
Mean ± SD	2.5 ± 0.9	8.1 ± 1.4	3.2 ± 1.5	8.1 ± 2.6	4.4 ± 2.1	13.2 ± 3.2

**Figure 5:**
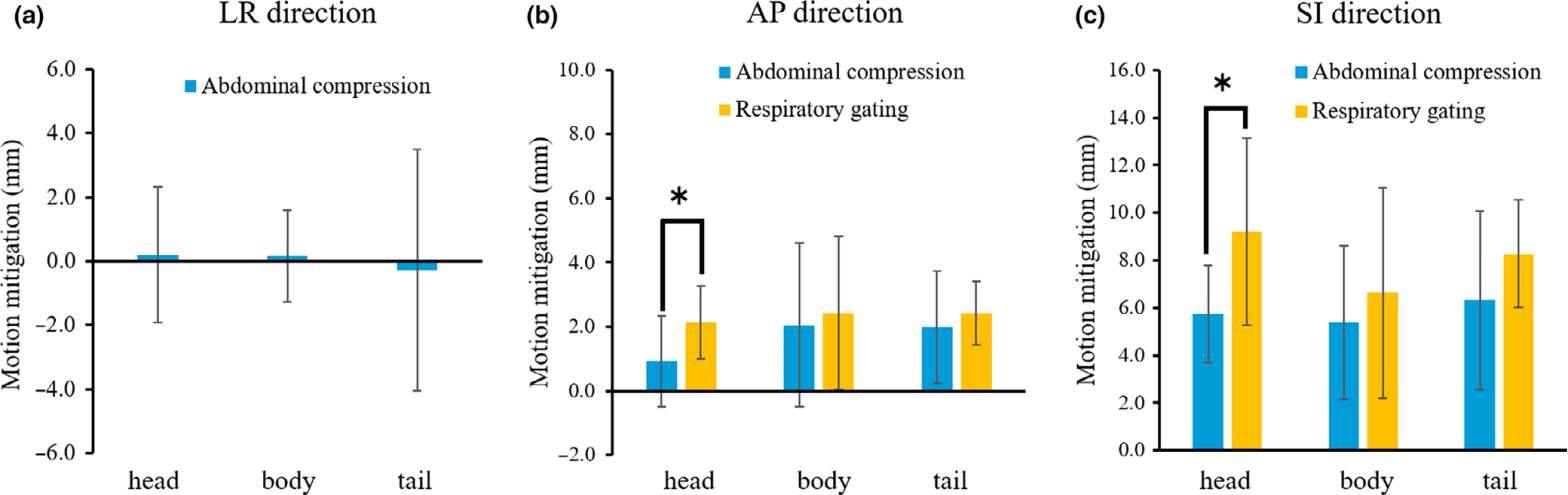
The magnitude of motion mitigation with abdominal compression and respiratory gating based on the abdominal wall surrogate in the left–right (LR) (a), anterior–posterior (AP) (b), and superior–inferior (SI) directions (c). *P < 0.05.

Figure 5 shows the magnitude of respiration‐induced motion mitigation for each pancreatic region during AC in the LR (a), AP (b), and SI directions (c) and during RG based on the abdominal wall surrogate in the AP (b) and SI directions (c). The LR direction showed an increase, although not significant, in respiration‐induced motion with AC, especially in the tail region. In the AP direction, the magnitude of motion mitigation in the head region was significantly greater with RG than it was with AC (*P* = 0.03), but none of the other regions showed significant differences in the magnitude of motion mitigation between RG and AC (body: *P* = 0.38, tail: *P* = 0.25). In the SI direction, the magnitude of motion mitigation in the head region was significantly greater with RG than it was with AC (*P* = 0.02), but the magnitude of motion mitigation between RG and AC was not significantly different in the other regions (body: *P* = 0.27, tail: *P* = 0.10). The mean ± standard deviation values for motion mitigation of the entire pancreas with AC in the LR, AP, and SI directions were 0.0 ± 2.5 mm, 1.7 ± 2.0 mm, and 6.0 ± 3.5 mm respectively. The corresponding values with RG in the AP and SI directions were 2.5 ± 1.6 mm and 7.7 ± 4.1 mm, respectively.

## 4 DISCUSSION

In this study, we found that the magnitude of pancreatic motion with or without AC and the magnitude of the positional error with RG varied depending on the pancreatic region. When comparing the effects of motion mitigation between AC and RG, the magnitude of motion mitigation in the AP and SI directions with RG was greater than that with AC in the pancreatic head region.

Our study evaluated the respiration‐induced motion of each pancreatic region with and without AC. The magnitude of pancreatic motion in the SI direction was assessed in both sagittal and coronal cine‐MRI (Table 1). Both revealed almost equivalent results for each pancreatic region with and without AC. As coronal cine‐MRI included each pancreatic region in the same plane, motion variations for each pancreatic region were simultaneously confirmed.

Several prior studies examined pancreatic tumor motion with or without AC and evaluated the efficacy of AC.^13^, ^18^, ^32^ Table 3 shows the studies that evaluated the motion mitigation values with AC in LR, AP, and SI directions. Heerkens et al.^18^ reported motion mitigation values of −0.4, 0, and 4.1 mm in the LR, AP, and SI directions, respectively, when using an abdominal corset. In our study, AC using an in‐house‐developed compression device reduced respiration‐induced motion in each pancreas region in the AP and SI directions, exhibiting similar trends as those observed in the previous reports. However, in the assessments of different pancreatic regions, some volunteers showed an increase in respiration‐induced motion in the LR direction with AC, especially in the tail region. Furthermore, regardless of AC application, the respiration‐induced motion in the tail region in the SI and LR directions was larger than that in the other regions. Campbell et al.^13^ evaluated CBCT images and compared the efficacy of RG using the abdominal wall surrogate with that of AC for reducing target motion. In their results, the mean pancreatic motion mitigation values with AC and RG were, respectively, 2.0 and 3.4 mm in the AP direction and 5.4 and 8.4 mm in the SI direction. Although these results are almost equivalent to our results, in our analyses of different pancreatic regions, there was a significant difference between the motion mitigation observed between RG and AC in both the AP and SI directions in the head region. However, in the tail region, no difference between the motion mitigation was achieved with RG vs. that achieved with AC was noted. These results may be attributable to the large positional error in the tail region in RG based on the abdominal wall surrogate (Fig. 4). Therefore, our results showed that RG using the abdominal wall surrogate is a more effective strategy than is AC for mitigating target motion in the pancreatic head region. In contrast, in the body and tail regions, RG and AC showed equivalent motion‐mitigating effects. Mampuya et al.^16^, ^17^ reported that AC increases the interfraction variation in lung tumor position and can affect the local control rate after stereotactic body radiotherapy for primary lung cancer. As shown in Table 3, our study observed motion mitigations with AC under fasting state, which were comparable to those of previous studies for pancreatic cancer patients. Furthermore, an individualized approach seems essential for radiotherapy using AC because these variations of motion mitigation were large among individuals. However, since our study did not include MR scanning performed at multiple times, reproducibility of the interfractional pancreas position with AC needs to be further investigated.

**Table 3:** Summary of the studies evaluated the pancreatic motion mitigation values with abdominal compression in (LR), anterior–posterior (AP), and superior–inferior (SI) directions.

Author	Methods	LR (mm)	AP (mm)	SI (mm)
Our study	Cine MRI	0.0 (−5.5 to 5.9)	1.7 (−2.7 to 6.5)	6.0 (1.1 to 13.9)
Heerkens et al.^18^	Cine MRI	−0.4 (−4.5 to 1.3)	0 (−0.6 to 1.9)	4.1 (−2.3 to 17.2)
Campbell et al.^13^	CBCT	1.0 (−0.6 to 1.3)	2.0 (1.6 to 5.1)	5.4 (3.1 to 18.4)
Dolde K et al.^32^	4D MRI	−2.9 to 0.7	3.1 to 3.3	2.6 to 8.8

MRI = magnetic resonance imaging, CBCT = cone beam computed tomography 4D = four dimensional.

**Figure 6:**
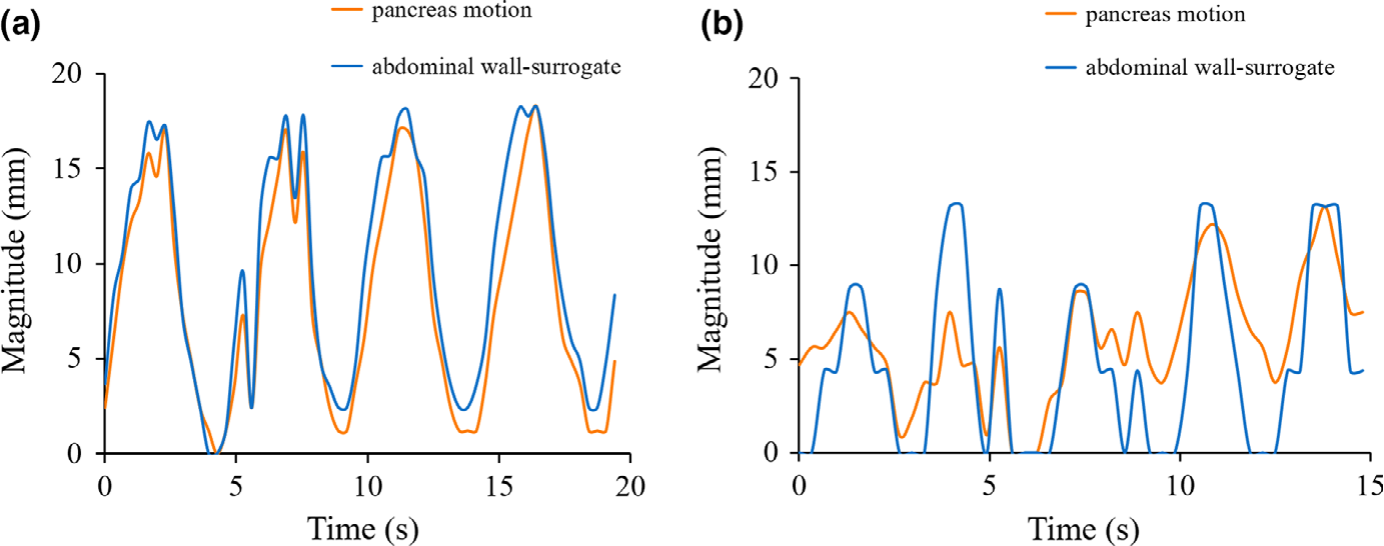
A typical example of the actual and predicted pancreas motion based on the abdominal wall surrogate in the SI direction at the same slice location of the pancreatic head region (volunteer no. 11). (a) With audio instruction. (b) Without audio instruction includes the respiration irregularities.

This study did not evaluate the respiration irregularities on the pancreatic motion because cine‐MR images were obtained under audio instructions. However, for volunteer no. 11, cine‐MR images without audio instructions were also obtained. Figure 6 shows the typical example of the actual and predicted pancreas motion based on the abdominal wall surrogate in the SI direction with or without audio instruction at the same slice location (head region). Several respiration irregularities were observed in each region of the pancreas on scanning without audio instructions. The mean values for the magnitude of positional error between actual and predicted pancreas motion in head, body, and tail regions on scanning with audio instructions were 1.0, 0.6, and 0.7 mm, respectively, and on scanning without audio instructions were 1.9, 1.5, and 1.9 mm respectively. In the treatment using RG with abdominal wall surrogate, respiration irregularities can increase the positional error and lead to unacceptably long treatment times. Heerkens et al.^25^ reported that gating schemes around the end expiration position seemed suboptimal for patients of pancreatic cancer who exhibited respiration irregularities. They also demonstrated that an individualized approach was essential for gated radiotherapy delivery in pancreatic cancer patients.

Previously, Huguet et al.^30^ reported that the correlation between the motion of fiducial markers and the pancreatic tumor motion was greater than the correlation between the motion of abdominal wall surrogate and pancreatic tumor motion. However, in the surrogate‐based treatment using fiducial markers, differences between the motion of the marker and that of other pancreatic regions or OARs adjacent to the pancreas might appear because of the region‐dependent pancreatic motion variation revealed in this study. As such, our findings should be considered during the treatment planning process when using small margins with gating or tracking strategies based on surrogate signals.

The direct tracking method for tumors in the current MRI‐guided radiotherapy system has the potential to minimize these errors.^15^ However, considering irradiation with sagittal‐based cine‐MRI guidance in the MRI‐guided radiotherapy system,^24^ our results showed that respiration‐induced motion in the LR direction perpendicular to the sagittal cine‐MR image was particularly large in the pancreatic tail region. Thus, it is necessary to evaluate three‐dimensional motion by using 4D MRI,^33^, ^34^ and individual respiration‐induced motion assessment is crucial.

As mentioned above, the present study assessed pancreatic region‐dependent variations in respiration‐induced motion and their effects on motion mitigation outcomes during AC or RG. Our results suggest that in clinical practice, where motion mitigation strategies such as AC or surrogate‐based RG or tracking are used, it is crucial to analyze the three‐dimensional tumor motion and the relationship between the surrogate signal and tumor motion for each individual patient, because the region‐dependent variations in pancreatic motion can affect the treatment accuracy. However, in our analysis of the magnitude of motion mitigation with RG based on the abdominal wall surrogate, it was not possible to evaluate pancreatic motion in the LR direction. In addition, as our study was limited to healthy volunteers, there is a need to evaluate the three‐dimensional motion of tumors in each pancreatic region with 4D MRI before adapting our findings to clinical practice. Furthermore, it is necessary to investigate which motion mitigation strategy is the most appropriate for specific conditions.

## 5 CONCLUSION

This study quantified the respiration‐induced motion of each pancreatic region during motion mitigation strategies and characterized the relationship between this motion and that of the surrogate signals using cine‐MRI. In radiation therapy of the pancreas, although motion mitigation strategies are effective, it is necessary to consider that the motion amplitude with or without AC and the magnitude of the predicted position error with RG will likely vary depending on the pancreatic region.

## CONFLICT OF INTEREST

There is no conflict of interest declared in this article.
